# Tolerability and effectiveness of albuvirtide combined with dolutegravir for hospitalized people living with HIV/AIDS

**DOI:** 10.1097/MD.0000000000035344

**Published:** 2023-11-10

**Authors:** Huanxia Liu, Shenghua He, Tongtong Yang, Chunrong Lu, Yuan Yao, Ruifeng Zhou, Ke Yin, Yuanhong He, Jing Cheng

**Affiliations:** a Clinical Section 1, Department of Infectious Disease, Public Health Clinical Center of Chengdu, Chengdu, China.

**Keywords:** advanced HIV, AIDS, albuvirtide, CD4+ T-cell, dolutegravir, HIV RNA

## Abstract

Treatment options for hospitalized people living with HIV/AIDS (PLWHA) with opportunistic infections and comorbidities are limited in China. Albuvirtide (ABT), a new peptide drug, is a long-acting HIV fusion inhibitor with limited drug-drug interactions and fast onset time. This single-center, retrospective cohort study investigated the effectiveness and safety of ABT plus dolutegravir (DTG) therapy in a real-world setting. We performed a chart review on the electronic patient records for hospitalized PLWHA using ABT plus DTG between April and December 2020. The clinical outcomes were retrospectively analyzed. Among 151 PLWHA (mean age 47.6 ± 15.9 years), 140 (93%) had at least 1 episode of bacterial and/or fungal infections and 64 (42%) had other comorbidities including syphilis, hepatitis B, and/or hypertension. ABT plus DTG was given to 87 treatment-naïve (TN) and 64 treatment-experienced (TE) PLWHA. Regardless of treatment history, mean HIV-1 RNA levels significantly decreased from 4.32 log_10_copies/mL to 2.24 log_10_copies/mL, 2.10 log_10_copies/mL and 1.89 log_10_copies/mL after 2, 4 and 8 weeks of treatment, respectively (*P* < .0001). Compared with baseline mean CD4 + T-cell counts of 122.72 cells/μL, it increased to 207.87 cells/μL (*P* = .0067) and 218.69 cells/μL (*P* = .0812) after 4 and 8 weeks of treatment. Except for limited laboratory abnormalities such as hyperuricemia, increased creatinine level, and hyperglycemia observed after treatment, no other clinical adverse events were considered related to ABT plus DTG. Data suggests that ABT plus DTG is safe and effective for critically-ill hospitalized PLWHA. In view of the rapid viral load suppression and restoration of CD4 + count within 8 weeks of treatment, its clinical application warrants further investigation.

## 1. Introduction

Treatment options are limited for hospitalized people living with human immunodeficiency virus (HIV)/acquired immunodeficiency syndrome (AIDS) with opportunistic infections and comorbidities. With high HIV-RNA levels and low CD4 + T-cell counts, people living with HIV/AIDS are at high-risk of advanced infections,^[1,2]^ which make them more susceptible to HIV infection- and treatment-related morbidities and thus increase mortality risk.^[3]^ Among people living with HIV/AIDS, wasting, the onset of opportunistic infections, chronic comorbidities, and drug toxicities can be causes for hospitalization.^[4]^

Anti-retroviral therapy (ART) has been proven effective in reducing viral load (VL), virulence, as well as morbidity and mortality of AIDS.^[5]^ However, there is a lack of robust evidence to support ART regimens use in individuals with critical-ill HIV disease. Critical-ill HIV-infected patients are admitted to the hospital for AIDS-related events or organ dysfunctions with critical care management.^[6]^ The decision to initiate or maintain highly active antiretroviral therapy in this situation is subjective, as there is no current consensus among experts.^[7]^ The ART choice for critically-ill populations with advanced HIV infection/AIDS are limited due to drug-drug interactions, overlapping toxicities, high pill burden and the possibility of immune reconstitution inflammatory syndrome.^[8–10]^ In China, efforts have been made to increase the coverage of ART for people living with HIV/AIDS.^[11–14]^ Nevertheless, poor treatment compliance and ART drug intolerance remained a concern and subsequently increasing treatment failure, emergence of drug resistance, suggesting an imminent need for alternative therapy.^[15]^

In this context, the dual regimen contains albuvirtide (ABT) plus dolutegravir (DTG). ABT is a chemically modified peptide that targets the HIV-1 gp41 envelope protein and inhibits the formation of the gp41 6-helix bundle structure (6-HB), which is necessary for the fusion of the viral and cellular membranes. ABT is a long-acting, once-weekly injectable HIV fusion inhibitor with few drug-drug interactions and a half-life of 11 to 12 days.^[16–18]^ ABT was proven to exhibit a broad spectrum of anti-HIV-1 activity, including viral strains predominantly circulating in China and some variants resistant to enfuvirtide (T20).^[19]^ In a prospective Phase II clinical study of anti-retroviral therapy (ART) – naïve subjects, the 2-drug regimen ABT plus ritonavir-boosted lopinavir (LPV/r) was associated with significant decreases in HIV-1 viral load.^[16]^ Through the Phase III TALENT study, ABT plus LPV/r was found non-inferior to standard WHO-recommended second-line LPV/r with 2NRTIs.^[20]^ Since ABT becomes available in China, real-world experience showed that it is well-tolerated with limited treatment-emergent adverse events and expanded uses were found especially for people who are of higher unmet needs and running out of treatment choices. Considering ART to combine with ABT, LPV/r may have several limitations including adverse events such as gastrointestinal intolerance and elevated lipid levels^[21]^ and potential drug-drug interactions with anti-fungal drugs, anti-tuberculosis drugs etc.^[22]^ DTG is now a first-line medication worldwide due to its high genetic barrier to resistance and superior efficacy.^[23]^ Another trial studied 28-days ABT plus dolutegravir (DTG) versus oral drug combinations of DTG, tenofovir disoproxil fumarate plus lamivudine (3TC) as post-exposure prophylaxis, and found the combination to be well-tolerated and effective with significantly greater adherence and completion rate than the oral drug regimen.^[24]^ Therefore, it is of interest to explore DTG, with the newly approved long-acting injectable fusion inhibitor for advanced, hospitalized HIV-1 infected patients. The present retrospective study aimed to investigate the clinical outcomes and safety of ABT plus DTG in a real-world setting.

## 2. Methods

A retrospective cohort study was conducted using electronic patient records from Chengdu Public Health Clinical Medical Center. This study was approved by the Ethics Committee of Chengdu Public Health Clinical Medical Center (Ethics Approval No.20180129) and the requirement for written consent was waived due to this study used routinely collected clinical data in an anonymized format.

**Figure 1.**
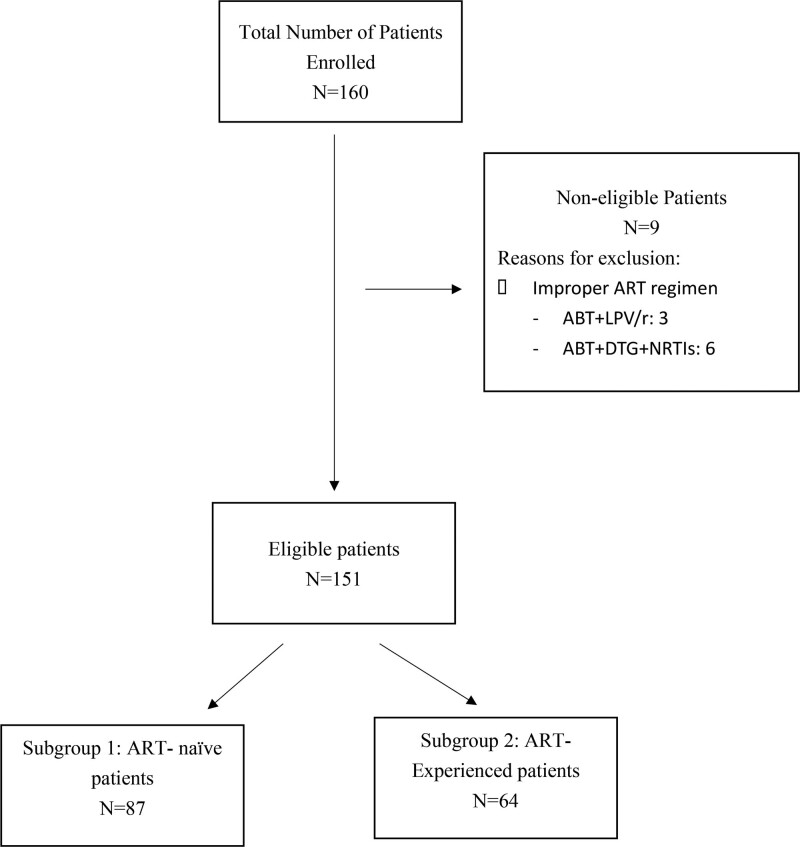
A flowchart shown the patient enrolled process. ART = anti-retroviral therapy, ABT = albuvirtide; LPV/r = ritonavir-boosted lopinavir, NRTIs = nucleoside/nucleotide reverse transcriptase inhibitors. Figure 1 shows how patients being enrolled to subgroup 1 (N = 87) and subgroup 2 (N = 64).

We reviewed ART records for hospitalized people living with HIV/AIDS (PLWHA) from April to December 2020 and included all patients at site who received ABT combined with DTG during hospitalization. Records with the following criteria were included in the analysis: people living with HIV/AIDS with opportunistic infections and/or exacerbation of comorbidities who were in critical condition; aged more than 18 years old; started antiviral treatment involving DTG 50mg once daily or twice daily and ABT 320mg IV once daily on day 1, 2, 3, and day 8 weekly onwards. Records were excluded for patients who were not tested for the plasma HIV-1 RNA and CD4 + T lymphocyte count after administration of ABT. A flowchart detailing the patient evaluation process was shown in Figure 1.

Clinical records of plasma HIV-1 RNA measured via Real-Time polymerase chain reaction and peripheral blood CD4-cell counts measured using a FACS count system before treatment, 2 weeks, 4 weeks and 8 weeks after treatment were retrospectively reviewed and analyzed. Toxicities were reviewed from the medical and laboratory records and were graded and evaluated according to Common Terminology Criteria for Adverse Events 5.0.^[25]^ Hospital discharge data was recorded and available, no long-term survival data was recorded in this study.

The baseline characteristics of patients were presented by descriptive analysis. Numerical data with a normal distribution are presented as mean and standard deviations (SDs) and those with non-normal distribution are summarized as medians and interquartile ranges. Analyses on the changes of HIV-1 RNA, CD4-cell counts and the ratio of CD4/CD8 along the treatment periods were performed using a mixed-effects model for repeated measures (MMRM) without imputation. Kenward-Rogers approach was used for degrees of freedom approximation. The Least Squares Means, 95% CI and *P* value of the change from baseline were reported by each visit, as well as *P* value of the average change from baseline of postbaseline versus baseline comparison. Log10 transformed data was applied in MMRM for HIV analysis, original data was applied in MMRM for CD4 and CD4/CD8 analysis. 95%CI for each visit was calculated based on Clopper-Pearson method, and 95%CI for the difference was calculated based on Miettinen and Nurminen (MN) method in analyzes for HIV RNA suppression in subgroup. The differences of viral load suppression rates between subgroups were analyzed by Chi-square tests. The results were considered statistically significant at *P* < .05. We used SAS version 9.4 (SAS Institute Inc., Cary, NC, USA) for all statistical analyses.

## 3. Results

Between April and December 2020, 151 critically-ill, hospitalized people living with HIV/AIDs treated with ABT plus DTG were retrospectively evaluated for the anti-viral effectiveness and treatment safety in clinical settings. Patients ranged from 21 to 87 (mean: 47.6 ± 15.9) years with over 55% of cases younger than 50 years old, and more than 80% being male. Among all patients in this study, 140 (93%) had at least 1 episode of bacterial and/or fungal infection and 64 (42%) had other comorbidities such as syphilis, hepatitis B, hypertension etc. A total of 87 (57.6%) patients were treatment-naïve (TN) and 64 (42.4%) were treatment-experienced (TE). Baseline characteristics are shown in Table 1.

**Table 1:** Participant characteristics (N = 151).

Characteristics	N (%)
Age	
≤50 yr old	83 (55.0%)
>50 yr old	68 (45.0%)
Gender	
Male	123 (81.5%)
Female	28 (19.4%)
Route of infection	
Injection drug use	1 (0.7%)
Heterosexual contact	32 (21.2%)
Homosexual contact	20 (13.2%)
Unknown	98 (64.9%)
Treatment history	
ART-naïve	87 (57.6%)
ART-experienced	64 (42.4%)
Reasons for drug initiation (in ART-naïve participants)	
Potential risk to DDI	83 (95.4%)
Anti-tuberculosis drugs	13 (14.9%)
≥3 types of anti-infection drugs due to complex infection	63 (72.4%)
Other concomitant medication	7 (8.1%)
Patient preference	4 (4.6%)
Reasons for drug adjustment (in ART-experienced participants)	
Drug resistance	12 (18.8%)
Virologic failure	29 (45.3%)
Potential risk to DDI due to severe OIs	23 (51.6%)
Previous ART	
None	87 (57.6%)
2 NRTIs + NNRTIs	41 (27.1%)
2 NRTIs + INSTIs	9 (6.0%)
2 NRTIs + PIs	14 (9.3%)
Co-morbidities	
None	87 (57.6%)
Infectious disease (including hepatitis, syphilis, etc)	33 (21.9%)
Chronic disease (including hypertension, diabetes, hyperlipidemia, etc)	31 (20.5%)
Opportunistic Infection	
None	11 (7.3%)
≤2 types of infection	66 (43.7%)
≥3 types of infection	74 (49.0%)
Concomitant Treatment for Infection	
None	8 (5.3%)
≤2 types of drugs	64 (42.4%)
≥3 types of drugs	79 (52.3%)
Baseline Laboratory	
Mean WBC in 10^9^/L (SD)	5.5 (3.0)
Mean ANC in 10^9^/L (SD)	4.1 (2.9)
Mean ALT in IU/L (SD)	59.4 (96.5)
Mean AST in IU/L (SD)	55.0 (72.7)
Baseline CD4 + T-cell, cell/μL	
>350	14 (9.3%)
>200–350	13 (8.6%)
>50–200	60 (39.7%)
≤50	64 (42.4%)
Baseline HIV-1 RNA, log_10_copies/mL	
Mean (SD)	4.32 (1.63)
Baseline CD4/CD8 ratio	0.28 (0.33)
ART regimen	
ABT exposure dose Mean (SD), mg	2574.83 (812.35)

ALT = alanine transaminase, ANC = absolute neutrophil count, ART = anti-retroviral therapy, AST = aspartate aminotransferase, DDI = drug-drug Interactions, INSTIs = integrase strand transfer inhibitors, IQRs = interquartile ranges, NNRTIs = non-nucleoside reverse-transcriptase inhibitors, NRTIs = nucleoside/nucleotide reverse transcriptase inhibitors, PIs = protease inhibitors, SD = standard deviation, WBC = white blood cell count.

Most patients had comorbidities or concomitant treatments (Table 1). ABT plus DTG was clinically initiated to avoid potential drug-drug interactions in 95.4% of ART-naïve and 51.6% of ART-experienced patients. In ART-naïve patients, the remaining 4.6% were treated with ABT plus DTG due to expressed preference. In ART-experienced patients, the reason for the switch to ABT plus DTG included virologic failure on previous line treatment (48.4%) (Table 1).

Regardless of treatment history, mean HIV-1 RNA levels significantly decreased from 4.32 log_10_copies/mL to 2.24 log_10_copies/mL, 2.10 log_10_copies/mL and 1.89 log_10_copies/mL after 2, 4 and 8 weeks of treatment, respectively (*P* < .0001) (Table 2, Panel A). Similarly, further analysis revealed a significant decreasing trend of HIV-1 RNA levels in both TN patients and TE patients (Table 3, Panel B). Mean CD4 + T-cell counts increased significantly from 122.72 cells/μL to 207.87 cells/μL after 4 weeks of treatment (*P* = .0067) but insignificantly to 218.69 cells/μL after 8 weeks of treatment (*P* = .0812) when compared to baseline. Further analysis revealed a significant increase of the mean CD4 + T-cell counts in TN patients from 90.48 cells/μL to 182.49 cells/μL and 222.99 cells/μL after 4 and 8 weeks of treatment (*P* < .0001), respectively. However, the increment of mean CD4 + T-cell counts in TE patients was significant from a baseline of 166.55 cells/μL to 221.60 cells/μL after 4 weeks of treatment (*P* = .0117) but insignificant to 203.79 cells/μL after 8 weeks of treatment (*P* = .1249). The mean CD4/CD8 ratio increased from a baseline of 0.28 to 0.31 after 4 weeks (*P* = .1617) and significantly to 0.38 after 8 weeks (*P* = .0008) of treatment in all patients. A statistically significant increase in the ratio was observed after both 4 and 8 weeks of treatment among TN patients and 4 weeks of treatment among TE patients.

**Table 2:** Summary of treatment outcomes in all study participants (Panel A) and sub-group analysis of 68 participants aged over 50 yr (Panel B).

A												
	All participants				Treatment-naïve participants				Treatment-experienced participants			
	Patient No.	Mean (SE)	Mean change from Baseline (SE)	*P* value*	Patient No.	Mean (SE)	Mean change from Baseline (SE)	*P* value*	Patient No.	Mean (SE)	Mean change from Baseline (SE)	*P* value*
HIV-1 RNA (log_10_copies/mL)												
Baseline	151	4.32 (0.13)			87	5.15 (0.11)			64	3.19 (0.20)		
2 wk	90	2.24 (0.06)	−2.09 (0.11)	<.0001	58	2.46 (0.08)	−2.69 (0.1)	<0.0001^*^	32	1.94 (0.08)	−1.25 (0.18)	<.0001
4 wk	83	2.10 (0.07)	−2.23 (0.13)	<.0001	57	2.30 (0.10)	−2.85 (0.13)	<0.0001^*^	26	1.82 (0.07)	−1.37 (0.20)	<.0001
8 wk	37	1.89 (0.08)	−2.43 (0.15)	<.0001	28	1.91 (0.10)	−3.24 (0.14)	<0.0001^*^	9	1.90 (0.20)	−1.29 (0.28)	<.0001
CD4 + T-cell (cells/μL)												
Baseline	151	122.72 (11.99)			87	90.48 (11.27)			64	166.55 (23.21)		
4 wk	95	207.87 (18.81)	85.15 (14.99)	.0067	62	182.49 (22.94)	92.01 (17.62)	<0.0001^*^	33	221.60 (28.95)	55.05 (20.72)	.0117
8 wk	44	218.69 (43.74)	95.96 (43.87)	.0812	30	222.99 (25.20)	132.51 (21.26)	<0.0001^*^	14	203.79 (27.34)	37.24 (23.72)	.1249
CD4/CD8 ratio												
Baseline	151	0.28 (0.03)			87	0.21 (0.03)			64	0.37 (0.05)		
4 wk	95	0.31 (0.03)	0.04 (0.03)	.1617	62	0.33 (0.04)	0.13 (0.03)	0.0002^*^	33	0.25 (0.03)	−0.12 (0.04)	.0060
8 wk	44	0.38 (0.04)	0.11 (0.03)	.0008	30	0.37 (0.05)	0.17 (0.04)	0.0001^*^	14	0.33 (0.04)	−0.04 (0.03)	.1854

B												
			Mean (SE)			Mean change from baseline (SE)			*P* value*			
HIV-1 RNA (log_10_copies/mL)												
Baseline (N = 68)			4.16 (0.20)									
2 wk (N = 35)			2.17 (0.09)			−1.99 (0.17)			<.0001			
4 wk (N = 34)			2.07 (0.09)			−2.09 (0.19)			<.0001			
8 wk (N = 15)			1.88 (0.13)			−2.28 (0.22)			<.0001			
CD4 + T-cell (cells/μL)												
Baseline (N = 68)			140.40 (17.59)									
4 wk (N = 40)			238.86 (28.55)			98.46 (22.63)			.0002			
8 wk (N = 17)			259.90 (37.42)			119.51 (38.08)			.0025			
CD4/CD8 ratio												
Baseline (N = 68)			0.34 (0.05)									
4 wk (N = 40)			0.37 (0.05)			0.03 (0.05)			.5269			
8 wk (N = 17)			0.47 (0.07)			0.13 (0.06)			.0431^*^			

* Compared to baseline.

**Table 3:** Achieving viral suppression following albuvirtide-based dual therapy, by baseline HIV-1 RNA levels (Panel A) and treatment status (Panel B).

A			
Duration of treatment	Viral Suppression with HIV-1 RNA < 40 copies/mL		*P* value‡
	Group A* N (%, 95%CI)	Group B† N (%, 95%CI)	
2 wk	53 (66.3%, 54.8–76.5%)	28 (39.4%, 28.0–51.8%)	.001
4 wk	60 (75.0%, 64.1–84.0%)	35 (49.3%, 37.2–61.4%)	.0011
8 wk	72 (90.0%, 81.2–95.6%)	59 (83.1%, 72.3–91.0%)	.2118

B			
Duration of treatment	Viral Suppression with HIV-1 RNA < 40 copies/mL		*P* value‡
	Treatment-naïve patients§ N (%, 95%CI)	Treatment-experienced patients∥ N (%, 95%CI)	
2 wk	37 (42.5%, 32.0–53.6%)	44 (68.8%, 55.9–79.8%)	.0014
4 wk	44 (50.6%, 39.6–61.5%)	51 (79.7%, 67.8–88.7%)	.0003
8 wk	72 (82.8%, 73.2–90.0%)	59 (92.2%, 82.7–97.4%)	.0912

* Group A (N = 80): Baseline HIV-1 RNA level <100,000 copies/mL.

† Group B (N = 71): Baseline HIV-1 RNA level ≥100,000 copies/mL.

‡ *P* value of Chi-square test was determined by comparing the percentage difference between groups.

§ Treatment-naïve patients (N = 87).

∥ Treatment-experienced patients (N = 64).

Sub-group analysis was performed to explore the change of VL and CD4 + T-cell counts among patients aged over 50. Similar results of significant reductions in HIV-RNA levels from 4.16 log_10_copies/mL to 1.88 log_10_copies/mL (*P* < .0001), as well as significant increases in CD4 + T-cell from 140.4 cells/μL to 259.9 cells/μL (*P* = .0025), and increasing CD4/CD8 ratio from 0.34 to 0.47 (*P* = .0431) after 8 weeks of treatment (Table 2, Panel B). Additional analysis was also conducted to examine the relationship between baseline HIV-1 RNA levels and viral suppression which was defined as HIV-1 RNA <40 copies/mL. After 8 weeks of treatment, 72 (90.0%) patients with baseline HIV-1 RNA levels of <100,000 copies/mL achieved viral suppression while 59 (83.1%) patients with higher baseline RNA levels of equal or more than 100,000 copies/mL achieved viral suppression (*P* = .2118) (Table 3, Panel A). Nevertheless, the difference became insignificant after 8 weeks of treatment. When comparing TN and TE patients, 59 (92.2%) TE patients achieved viral suppression of <40 copies/mL after 8 weeks of treatment compared to 72 (82.8%) TN patients albeit statistically insignificant (*P* = .0912) (Table 3, Panel B). According to baseline VL, there were 129 patients with baseline HIV-RNA ≥ 40 copies/mL and 22 patients with baseline undetectable VL (HIV-RNA < 40 copies/mL). After 8 weeks of treatment, mean HIV-RNA levels were reduced significantly from 4.79 log_10_copies/mL to 1.89 log10copies/mL (*P* < .0001), while mean CD4 + T-cell count was increased significantly from 97.86 cells/μL to 221.52 cells/μL (*P* < .0001) among the group of patients with detectable baseline VL (Table 4, Panel A). During the 8 weeks of treatment period, viral suppression was maintained among the group of patients with undetectable baseline VL (Table 4, Panel B).

**Table 4:** Viral load and CD4 count changes of study participants with Baseline HIV-1 RNA ≥ 40 copies/mL (Panel A) or <40 copies/mL (Panel B) after 4 wk and 8 wk of treatment.

A			
Duration of Treatment (Patients, No.)	HIV-1 RNA (log_10_copies/mL)		
	Mean (SE)	Mean change from baseline (SE)	*P* value*
Baseline (N = 129)	4.79 (0.11)		
2 wk (N = 82)	2.34 (0.06)	−2.44 (0.10)	<.0001
4 wk (N = 87)	2.12 (0.08)	−2.61 (0.12)	<.0001
8 wk (N = 45)	1.89 (0.09)	−2.90 (0.13)	<.0001

	CD4 + T cell count (cells/uL)		
	Mean (SE)	Mean change from baseline (SE)	*P* value*
Baseline (N = 129)	97.86 (9.26)		
4 wk (N = 88)	186.10 (17.59)	88.24 (13.90)	<.0001
8 wk (N = 40)	221.52 (20.38)	123.66 (17.49)	<.0001

B			
Duration of Treatment (Patients, No.)		Rate of HIV-1 RNA suppression (Plasma HIV-1 RNA < 40copies/mL)	
Baseline (N = 22)			
2 wk (N = 10)		100%	
4 wk (N = 9)		100%	
8 wk (N = 5)		100%	

* Compared to baselines.

No injection site reaction and ABT-related clinical adverse events were recorded among all patients during hospitalization, nor was there any self-reported complaint recorded during the treatment. Based on the analysis of laboratory tests of patients, the 3 most common adverse events included hyperuricemia, increased creatinine level and hyperglycemia after 4 and 8 weeks of ABT-dual therapy (Table 5). All hematologic and biochemical abnormalities were manageable. No ABT-emergent discontinuation cases were reported. Grade 5 adverse events were reported for 9 patients, all of them related to the outcome of opportunistic infections and not to the study drug.

**Table 5:** Incidence of laboratory abnormalities in participants receiving albuvirtide-based dual therapy after week 4 and week 8.

	After 4 wk of treatment (N = 115–118)*	After 8 wk of treatment (N = 52–54)*
	Incidence of grade level increase ≥ 1 N (%)	Incidence of grade level increase ≥ 1 N (%)
Hyperuricemia	26/116 (22.4%)	21/53 (39.6%)
Increased Creatinine	33/116 (28.5%)	20/53 (37.7%)
Hyperglycemia	28/115 (24.4%)	10/52 (19.2%)
Increased ALT	26/116 (22.4%)	8/52 (15.4%)
Increased AST	14/116 (12.1%)	8/52 (15.4%)
Increased ALP	9/115 (7.8%)	3/52 (5.8%)
Thrombocytopenia	19/118 (16.1%)	8/54 (14.8%)
Hypoalbuminemia	25/116 (21.6%)	4/52 (7.7%)
Anemia	26/118 (22.0%)	4/54 (7.4%)
Leukopenia	2/118 (1.7%)	3/54 (5.7%)
Neutropenia	1/118 (0.9%)	1/54 (1.9%)
Hypoglycemia	5/115 (4.4%)	–

ALP = alkaline phosphatase, ALT = alanine aminotransferase, AST = aspartate aminotransferase.

* Not all participants have full set of blood assessment.

Despite the lack of detailed records of other adverse events, 142 out of 151 patients were stable and discharged from the hospital after ABT-dual therapy with a mean length of hospitalization of 29.3 ± 13.1 days. The majority patients switched back to oral ART at discharge.

## 4. Discussion

Managing advanced hospitalized HIV/AIDS patients are challenging, especially since they are comorbid with different opportunistic infections and comorbidities. There is no “one-size-fits-all” ART treatment for critically ill patients. The result of our study revealed that the virological response and immune recovery of critically ill people living with HIV/AIDS after the use of ABT in combination with DTG, and that this ART regimen was found to be safe and well tolerated.

A significantly rapid viral suppression was observed in all critically-ill patients treated with ABT plus DTG as well as among TE or TN patients after 4 and 8 weeks of treatment. Within 8 weeks of ABT plus DTG therapy, 90% of patients with baseline HIV-1 RNA levels <100,000 copies/mL and 83.1% of patients with baseline HIV-1 RNA levels more than 100,000 copies/mL both achieved HIV-1 RNA levels below 40 copies/mL. When comparing treatment history, both TN (82.8%) and TE patients (92.2%) achieved viral suppression after 8 weeks of treatment. ABT plus DTG was highly effective in TE patients either with undetectable baseline viral load or virological failure. The results are consistent with the TALENT study, 75.7% of study participants treated with ABT/LPV/r achieved HIV-1 RNA levels below 40 copies/mL after 48 weeks of treatment.^[26]^ In another multicenter cohort study to assess DTG-based regimens in advanced HIV-infected naïve patients, the result demonstrated HIV-1 RNA < 40 copies/mL was achieved in 36% and 60% of individuals on DTG-based regimens at 1 month and 3 months, respectively.^[27]^ High pretreatment HIV-RNA levels are also considered an independent predictor of delayed virological suppression, increased risk of virological failure, and subsequently increased risk of mortality.^[28–33]^ In this retrospective study, 83.1% of study participants with a high baseline HIV-RNA level of more than 100,000 copies/mL had undetectable VL after 8 weeks of ABT treatment. Encouraging results as the complete virological suppression was achieved within only several weeks of treatment and patients were discharged from the hospital. These results showed virological suppression in critically ill individuals regarding baseline viral load administrated with ABT + DTG was comparable with other DTG or ABT-based regimens.

Another key finding is the significant immune recovery after 8-week treatment of ABT + DTG. The result is more prominent in TN patients. The mean CD4 + T-cell counts increased by > 100 cells/uL after 8 weeks of treatment from baseline. Another study by Yang J. et al also reported an increased trend of CD4 + cell count in treatment naïve patients using ABT monotherapy for 30 days.^[34]^ Highly active antiretroviral therapy can induce sustained recovery of CD4 + T cell reactivity against opportunistic pathogen in severely immunosuppressed patients.^[35]^ Decreasing viral load can reverse HIV-driven activation and CD4 + T cell defects in advanced HIV-infected patients.^[36]^ While promising effectiveness was observed in this retrospective study, a few potential prognostic predictors were examined. A low CD4/CD8 ratio was considered predictive of poor prognosis with non-AIDS related events or death even during effective ART.^[37,38]^ In our study, the CD4/CD8 ratio did not increase significantly among participants previously treated with ART, although the ratio slightly improved after 8 weeks of treatment, which might reflect lower immunological recovery for TE individuals than TN people living with HIV/AIDS. As this study did not provide data on optimal treatment duration beyond 8 weeks, additional research is needed to determine the disease prognosis after a longer treatment duration.

In our study, ABT plus DTG were well tolerated in this group of critical-ill patients living with HIV/AIDS, reflected by no adverse reactions related to the study drug and no ABT-emergent discontinuation cases. WHO recommends patients with advanced HIV should have rapid ART initiation (except those complicated with cryptococcal meningitis).^[39]^ Good tolerability and low drug-drug interactions are critical considerations for treatment selection.

This retrospective cohort study investigated the use of ABT plus DTG dual therapy in a real-world setting. The treatment outcome and safety profile should be carefully interpreted and several limitations should be noted. This study does not answer the question on the efficacy and safety of ABT plus DTG versus standard-of-care treatments because this was a single-arm study. Data of patients was only recorded up to 8 weeks or shorter and for routine clinical care without long-term follow-up. There is a need for a well-controlled study to investigate which populations may derive the optimal benefit of ABT-based therapy, as well as the optimal treatment duration for ABT plus DTG to achieve and prolong therapeutic outcomes. An extended review on the survival outcomes of patients treated with ABT may help determine the long-term treatment impact. Such investigations could also include the efficacy of ABT in individuals with T20 peptide resistance.

## 5. Conclusion

This retrospective cohort study showed that ABT plus DTG therapy in clinical practice provided a high rate of viral suppression and restoration of CD4 + T cell count within 8 weeks of treatment. No significant safety concerns were observed. Therefore, in the short term, the ABT-based regimen is well tolerated, and the viral load quickly becomes undetectable, but it remains to be investigated if this is maintained over time.

## Acknowledgments

The authors disclosed receipt of the following financial support for the research, authorship, and/or publication of this article: This work was supported by the Health Planning Committee Project Foundation of Sichuan [Grant numbers 18PJ341].

## Author contributions

**Investigation:** Huanxia Liu, Shenghua He, Tongtong Yang, Chunrong Lu, Yuan Yao, Ruifeng Zhou, Ke Yin, Yuanhong He, Jing Cheng.

**Methodology:** Huanxia Liu.

**Project administration:** Huanxia Liu.

**Supervision:** Shenghua He.

**Writing – original draft:** Huanxia Liu.

**Writing – review & editing:** Shenghua He.
